# The complete mitochondrial genome of *Gracilaria textorii* (Gracilariales, Florideophyceae)

**DOI:** 10.1080/23802359.2018.1457990

**Published:** 2018-04-03

**Authors:** Xiaochu Yuan

**Affiliations:** College of Marine Life Sciences, Ocean University of China, Qingdao, China

**Keywords:** *Gracilaria textorii*, mitochondrial genome, phylogenetic analysis

## Abstract

In this study, we determined the mitochondrial genome sequence of *Gracilaria textorii*. The mitogenome of *G. textorii* is 25,743 bp in length, with GC content of 28.2%, and includes 25 protein-coding genes, two rRNA genes (*rns* and *rnl*), and 22 tRNA genes. The overall nucleotide composition of *G. textorii* includes 33.9% A, 14.1% C, 14.1% G, and 28.2% T. The newly sequenced mitogenome data were used for reconstruction of phylogenetic relationships in red algae, and the results suggested that *G. textorii* was closely related to *G. salicornia*.

*Gracilaria textorii* was first described from Japan by Suringar in 1867 under the name *Sphaerococcus (Rhodymenia) textorii*. In 1895, De Toni transferred it to the genus *Gracilaria,* newly named *Gracilaria textorii* (Sur.) (Ohmi 1955; Chang and Xia 1964). The specie usually grows on rocks and stones in the low tide zone or below the low-water mark. In China, it is mainly common along the coast of Liaoning Province, Shandong Province, and Guangdong Province. *Gracilaria* species are used worldwide in the production of agar (Armisen 1995). The current research on *G. textorii* has mostly focused on the extraction of polysaccharides and exploration of marine drug substances (Oza et al. 2011), while studies on molecular biology of *G. textorii* are still scarce.

In this study, we first obtained the mitochondrial genome sequence of *G. textorii* that was collected from Rizhao, Shandong Province, China (119°35′58″E, 35°27′54″N), and stored in the Culture Collection of Seaweed at the Ocean University of China (sample accession number: 2017050065). Total DNA was extracted from ∼1 g of frozen tissue using the modified CTAB method (Sun et al. 2011) and whole genome sequencing was performed by the next-generation sequencing. *Gracilaria salicornia* mitogenome (NC_023784) was aligned as a reference to assemble the complete mitogenome of *G. textorii*. The protein-coding genes and ribosomal RNA genes of *G. textorii* were annotated based on *G. salicornia* using Geneious R 10 (http://www.geneious.com/). To predict the tRNA genes, the mitochondrial genome sequences were submitted to the tRNAscan-SE v. 1.21 (Lowe and Eddy 1997). The physical map of the mitogenome was prepared for visualization using Organellar Genome DRAW (OGDraw) (Lohse et al. 2007).

The complete mitochondrial genome of *G. textorii* is a circular molecule that is 25,743 bp in length (GenBank accession number: MG592729), has an overall GC content of 28.2%, and encodes 49 genes, of which 24 are protein-coding genes with one open reading frame (*orf148*), two are rRNA genes (*rns* and *rnl*), and 22 are tRNA genes. The nucleotide composition of *G. textorii* is as follows: 33.9% A, 14.1% C, 14.1% G, and 28.2% T. Non-coding region comprises 7.38% of the mitogenome. The length of the protein-coding region is 18,147 bp, accounting for 70.49% of the total mitogenome size. The tRNA genes range from 72 to 93 bp in length, and they are evenly encoded by both the H- and L-strand. All protein-encoding genes start with an ATG codon and TAA and TAG are stop codons; TAG and TAA reach 8% and 92%, respectively. The genomic structure appeared to be highly conserved in Gracilariaceae.

In order to reconstruct the evolutionary relationships in Gracilariaceae, we used Bayesian method to build the phylogenetic tree, using the following parameters: two independent run with four Markov chains Monte Carlo runs, each of 1,000,000 generations, until the *p* value was less than .01; 25% aging samples were discarded in burn-in. *Cyanidioschyzon merolae* was selected as an outgroup. The resulting topology (Figure 1) offered molecular basis for its taxonomy, and indicated *G. textorii* was closely related to *G. salicornia* within Gracilariaceae.

**Figure 1.**
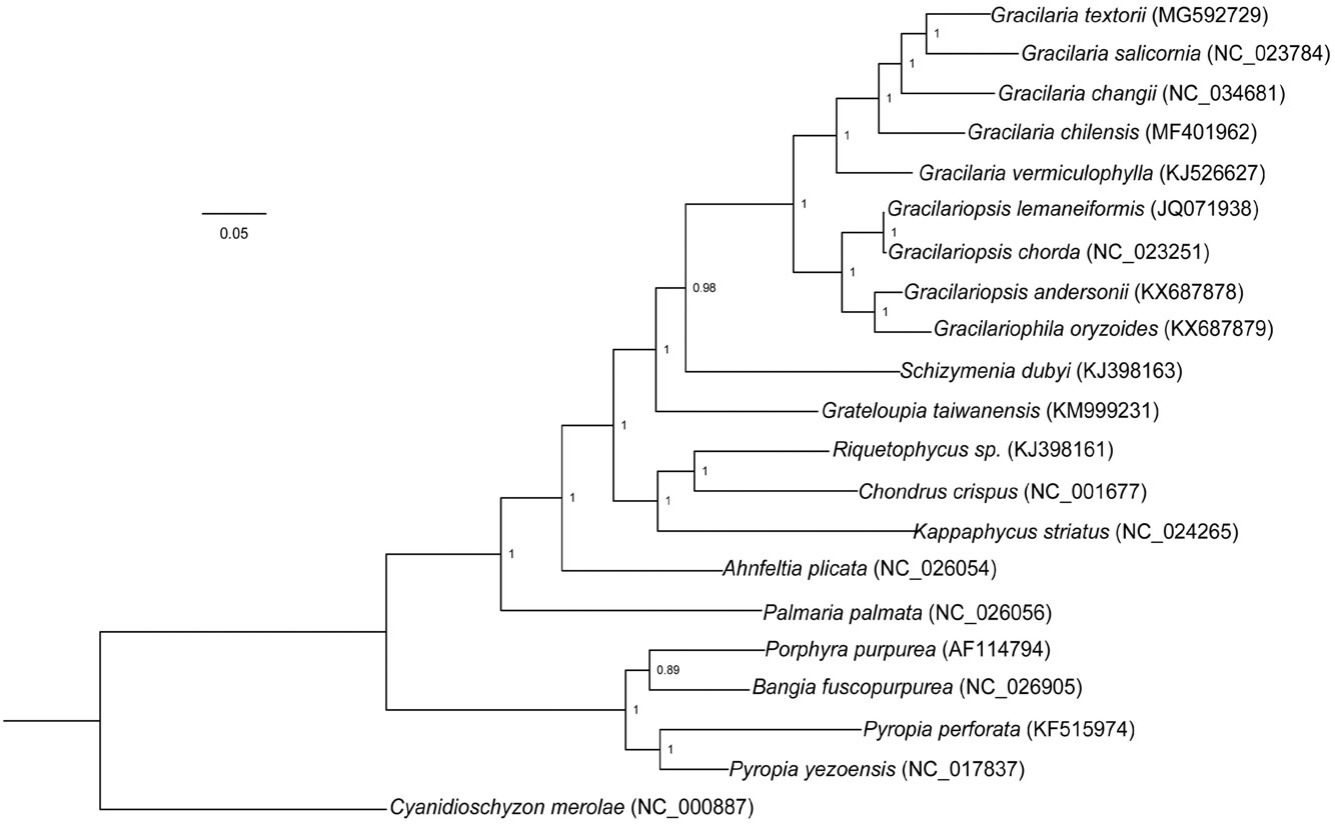
Bayesian phylogenetic tree was constructed based on the amino acid dataset of 24 protein genes. All red algae species are listed as follows: *Ahnfeltia plicata* (NC_026054), *Bangia fuscopurpurea* (NC_026905), *Chondrus crispus* (NC_001677), *Gracilaria chilensis* (NC_026831), *Gracilaria salicornia* (NC_023784), *Gracilaria vermiculophylla* (KJ526627), *Gracilaria changii* (NC_034681), *Gracilariophila oryzoides* (KX687879), *Gracilariopsis andersonii* (KX687878), *Gracilariopsis chorda* (NC_023251), *Gracilariopsis lemaneiformis* (JQ071938), *Grateloupia taiwanensis* (KM999231), *Kappaphycus striatus* (NC_024265), *Palmaria palmata* (NC_026056), *Porphyra purpurea* (AF114794), *Pyropia yezoensis* (NC_017837), *Pyropia perforata* (KF515974), *Riquetophycus sp.* (KJ398161), *Schizymenia dubyi* (KJ398163), and *Cyanidioschyzon merolae* (NC_000887).

## References

[CIT0001] Armisen R. 1995 World-wide use and importance of *Gracilaria*. J Appl Phycol. 7:231.

[CIT0002] Chang CF, Xia BM. 1964 A comparative study of *Gracilaria foliifera* (forssk.) bφrgs. and *Gracilaria textorii* (suring.) detoni. Acta Bot Sin. 12:201–209.

[CIT0003] Lohse M, Drechsel O, Bock R. 2007 OrganellarGenomeDRAW (OGDRAW): a tool for the easy generation of high-quality custom graphical maps of plastid and mitochondrial genomes. Curr Genet. 52:267–274. 17957369 10.1007/s00294-007-0161-y

[CIT0004] Lowe TM, Eddy SR. 1997 tRNAscan-SE: a program for improved detection of transfer RNA genes in genomic sequence. Nucleic Acids Res. 25:955–964. 9023104 10.1093/nar/25.5.955 PMC146525

[CIT0005] Ohmi H. 1955 Contributions to the knowledge of Gracilariaceae from Japan. I: critical notes on the structure of *Gracilaria textorii* (suringar) j. ag. Bull Faculty Fish Hokkaido Univ. 5:320–331.

[CIT0006] Oza MD, Mehta GK, Kumar S, Meena R, Siddhanta AK. 2011 Galactans from *Gracilaria millardetii* and *Gracilaria textorii* (Gracilariales, Rhodophyta) of Indian waters. Phycol Res. 59:244–249.

[CIT0007] Sun YY, Luo D, Zhao C, Li W, Liu T. 2011 DNA extraction and PCR analysis of five kinds of large seaweed under different preservation conditions. Mol Plant Breed. 9:1680–1691.

